# Atezolizumab plus nab-paclitaxel for unresectable, locally advanced or metastatic breast cancer: real-world results from a single academic center in Austria

**DOI:** 10.1186/s12885-022-10168-4

**Published:** 2022-10-26

**Authors:** Christine Deutschmann, Rupert Bartsch, Christian F Singer, Daphne Gschwantler-Kaulich, Michael Seifert, Carmen Leser, Maximilian Marhold, Zsuzsanna Bago-Horvath, Georg Pfeiler

**Affiliations:** 1 Department of Obstetrics and Gynecology, Division of General Gynecology and Gynecologic Oncology, Medical University of Vienna, Waehringer Guertel 18-20, 1090 Vienna, Austria; 2 Department of Medicine 1, Clinical Division of Oncology, Medical University of Vienna, Vienna, Austria; 3 Department of Pathology, Medical University of Vienna, Vienna, Austria

**Keywords:** Breast cancer, Atezolizumab, Real-world, Progression-free survival, Overall survival

## Abstract

**Purpose:**

IMpassion130 led to the approval of atezolizumab plus nab-paclitaxel as first-line treatment for patients with unresectable locally advanced or metastatic triple-negative, PD-L1 immune-cell positive breast cancer (BC) by the European Medicines Agency (EMA). The objective of the present study was to investigate the implementation, safety and efficacy of this combination in the initial phase after approval.

**Methods:**

A retrospective data analysis including all BC patients who received atezolizumab and nab-paclitaxel between 1.1.2019 and 31.10.2020 at the Department of Obstetrics and Gynecology and the Department of Medicine 1, respectively, at the Medical University of Vienna, Austria, was performed. Progression-free survival (PFS) and overall survival (OS) were estimated with the Kaplan-Maier product-limit method. Owing to the retrospective nature of this study, all statistics must be considered exploratory.

**Results:**

In total 20 patients were included in the study. Median follow-up was 7.1 months (IQR 5.2–9.1). Median PFS was 3.0 months (SE = .24; 95% CI [2.5; 3.5]). Median OS was 8.94 months (SE = 2.34, 95%CI [4.35; 13.53]). No new safety signals were observed.

**Conclusion:**

The present study showed a considerably shorter PFS (3.0 vs. 7.5 months) and OS (8.94 vs. 25.0 months) than IMpassion130 putatively owing to the use of atezolizumab in later treatment lines, more aggressive tumors and a study population with higher morbidity compared to the pivotal trial.

## Introduction

The phase 3 IMpassion130 study compared atezolizumab, a monoclonal PD-L1 antibody, plus nab-paclitaxel to placebo plus nab-paclitaxel as first-line treatment for patients with unresectable locally advanced or metastatic triple-negative breast cancer (TNBC) [1]. The median progression-free survival (PFS) was significantly improved with atezolizumab plus nab-paclitaxel compared to placebo plus nab-paclitaxel (7.5 months (95%CI 6.7–9.2) vs. 5.0 months (95%CI 3.8–5.6); hazard ratio [HR] 0.62, 95% CI 0.49–0.78; *p* < 0.0001) in the PD-L1 immune-cell positive subgroup (tumors that express PD-L1 on immune cells that cover 1% or more of the tumor area). In the exploratory overall survival analysis of patients with PD-L1 immune cell-positive tumors, median OS was 25.0 months with atezolizumab plus nab-paclitaxel and 15.5 months with placebo plus nab-paclitaxel (hazard ratio, 0.62; 95%CI, 0.45 to 0.86). These results led to the approval of atezolizumab plus nab-paclitaxel in the aforementioned patient population by the European Medicines Agency (EMA).

The second interim overall survival analysis of IMpassion130 after a median follow-up of 18.5 months (IQR 9.6–22.8) in the atezolizumab group and 17.5 months (IQR 8.4–22.4) in the placebo group confirmed previous findings.

In general, atezolizumab and nab-paclitaxel demonstrated an acceptable safety profile, yet adverse events of special interest have to be considered.

Patients in clinical trials – especially in those investigating new medical treatments – are highly selected and stringent study-specific in- and exclusion criteria often cannot be met in a real-world setting. Yet, it is an ethical dilemma to withhold a potential effective treatment option from a metastatic cancer patient solely owing, for instance, to the treatment line. Therefore, it is very likely that the real-world patient population does not completely match with the underlying study population. Consequently, it is important to review the efficacy and safety of a new therapy option in the real-world population.

The primary objectives of the present study were to describe the real-world patient population receiving atezolizumab and nab-paclitaxel at an academic center in Austria as well as to evaluate treatment efficacy outcome parameters. The secondary objective was to assess the treatments´ safety profile in this patient population.

## Patients and methods

A retrospective data analysis including all unresectable locally advanced or metastatic breast cancer patients who received atezolizumab and nab-paclitaxel between 1.1.2019 and 31.10.2020 at the Department of Obstetrics and Gynecology and the Department of Medicine 1, respectively, at the Medical University of Vienna, Austria was performed.

Patients were assigned to atezolizumab and nab-paclitaxel by an interdisciplinary tumor conference. Concordantly to IMpassion130, patients had received atezolizumab 840 mg intravenously on day 1 and day 15 of every 28-day cycle and nab-paclitaxel 100 mg/m^2^ of body surface area intravenously on days 1, 8 and 15 until progression, unacceptable toxicity or death. Dose adjustments were performed when indicated.

PD-L1 status was assessed using the Ventana SP142 kit in 16 samples; in 2 samples the Clone BSR90 (Nordic Biosite) was used. In 4 samples, the respective test kit was not stated.

Laboratory tests were performed according to local routine. Adverse events were assessed prior to every treatment administration in the course of the routine clinic visits on days 1, 8 and 15.

Baseline staging evaluation consisted of breast imaging (mammography, breast ultrasound, or breast MRI, as indicated), CT scans of chest and abdomen, with further work-up if required. Restaging investigations were performed approximately every three months.

Progression free survival (PFS) and time to treatment failure (TTF) were assessed radiologically and by clinical examination.

Statistical analysis was performed with IBM SPSS Statistics Version 22. A *p*-value of ≤ 0.05 was considered significant.

Due to the retrospective nature of this study, all statistics must be considered exploratory.

## Results

A total of 20 patients received atezolizumab and nab-paclitaxel for advanced breast cancer between 1.1.2019 and 31.10.2020 at the Department of Obstetrics and Gynecology and the Department of Medicine 1, respectively, at the Medical University of Vienna, Austria.

Of these patients, one male patient was diagnosed with a cancer of unknown primary most likely deriving from a triple-negative breast cancer. A biopsy of an axillary lymph node metastasis showed a triple negative receptor expression status, while a biopsy of a peritoneum metastasis had a weak positive progesterone receptor expression.

Another patient was first diagnosed with a luminal B primary breast tumor in March 2016. In April 2017 she presented with triple negative skin metastases. In May 2017 she was diagnosed with lung metastases and in November 2017 with liver metastases which were not biopsied. In July 2018 she was diagnosed with an ipsilateral triple negative breast tumor.

One patient was first diagnosed with a luminal B primary breast cancer in June 2013. In November 2014 she presented with bone metastases which were not biopsied. In April 2016 she was diagnosed with liver metastases which were repeatedly biopsied in September 2018, September 2019 and October 2019 and showed variable immunohistochemistry profiles (some lesions had a positive ER and PR expression profile, others a triple negative receptor expression profile).

All other patients had solely biopsies of triple negative primary breast tumors and according metastatic lesions.

Details regarding patient and tumor characteristics are outlined in Table 1.

**Table 1:** Characteristics of patients/disease at baseline

	**IMpassion130**, Intention-to-treat-population, atezolizumab + nab-paclitaxel (*n* = 451)	**IMpassion130**, PD-L1 positive subgroup, atezolizumab + nab-paclitaxel (*N* = 185)	**Deutschmann C**, et al. (*N* = 20)
**Age, years**	55 (46–64)	53 (44–63)	53 (40–60)
Distribution – no. (%)			
18–40	63 (14%)	31 (17%)	6 (30%)
41–64	284 (63%)	111 (60%)	10 (50%)
≥ 65	104 (23%)	43 (23%)	4 (20%)
**Sex**			
Female	448 (99%)	184 (99%)	19 (95%)
Male	3 (1%)	1 (1%)	1 (5%)
**Baseline disease status**			
Metastatic	404/450 (90%)	162/185 (88%)	19 (95%)
**Number of metastatic sites**			
0–3	332/450 (74%)	149/185 (81%)	15 (75%)
≥ 4	118/450 (26%)	36/185 (19%)	5 (25%)
**Site of metastatic disease**			
Liver	126 (28%)	44 (24%)	8 (40%)
Bone	145 (32%)	54 (29%)	6 (30%)
Brain	30 (7%)	15 (8%)	4 (20%)
asymptomatic, treated			1 (5%)
symptomatic, treated			3 (15%)
Lung	226 (51%)	86 (46%)	11 (55%)
Lymph node only	33/450 (7%)	18/185 (10%)	0
**Previous neoadjuvant or adjuvant treatment**	284 (63%)	125 (68%)	15 (75%)
**Previous taxane use**^**a**^	231 (51%)	96 (52%)	14 (70%)
**Previous anthracycline use**^**a**^	243 (54%)	109 (59%)	14 (70%)
**Previous radiotherapy**^**a**^	268 (59%)	119 (64%)	12 (60%)
**Radiotherapy of the brain**	25 (6%)	14 (8%)	3 (15%)
**Time from last surgery until diagnosis with unresectable locally advanced or metastatic disease, months**^**c**^	24.5 (15.9–38.9)	21.5 (15.0–36.2)	9.0 (6.2–22.5)
**Treatment line for metastatic disease**			
I			9 (45%)
II			3 (15%)
III			4 (20%)
IV			2 (10%)
V			2 (10%)
**Number of subsequent treatment lines following atezolizumab (and nab-paclitaxel) discontinuation at the time of data cut-off**			
1			4 (22%)
2			6 (33%)
3			2 (11%)
Unknown			2 (11%)
**PD-L1 status**			
PD-L1 positive			17 (85%)
PD-L1 negative			2 (10%)
**PD-L1 biopsy site**			
Breast	576 (63.9%)^d^		9 (45%)
Lymph nodes^b^	109 (12.1%)^d^		5 (25%)
Skin	21 (2.3%)^d^		6 (30%)
Liver	47 (5.2%)^d^		1 (5%)
Peritoneum	Unknown		1 (5%)
Brain	9 (1%)^d^		2 (10%)
Lung	54 (6%)^d^		0
Soft tissue	39 (4.3%)^d^		0
Other	46 (5.1%)^d^		0

^a^In curative treatment setting

^b^Including axillary and supraclavicular lymph nodes

cIncluding only patients with secondary metastasized breast cancer

^d^Extracted from the exploratory biomarker evaluation of the IMpassion130 study

Five patients presented with de novo and 15 patients with secondary metastasized breast cancer.

Median follow-up in the entire population was 7.1 months (IQR 5.2–9.1). The median time between the date of tissue sampling that was (later) used for PD-L1 assessment and the start date of atezolizumab and nab-paclitaxel treatment was 6.6 months (IQR 1.1–12.4).

Two tissue samples in the present study were PD-L1 negative. One PD-L1 negative tissue sample (unknown localization) was obtained from a patient that also had a PD-L1 positive brain metastasis. In the other respective sample obtained from a male patient with a cancer of unknown primary most likely deriving from a triple-negative breast cancer the PD-L1 status of the tumor-infiltrating lymphocytes was negative, but 2% of the tumor cells were PD-L1 positive. The patient was informed about the experimental character of the treatment with atezolizumab and nab-paclitaxel based on the negative PD-L1 status of the immune cells.

In four patients PD-L1 status was assessed in more than one tissue sample. Three patients showed concordantly positive PD-L1 results (In two of these patients PD-L1 status was determined in the primary breast tumor and a skin metastasis and in one patient in the primary breast tumor and a supraclavicular lymph node metastasis, respectively), while one patient had a PD-L1 positive axillary lymph node metastasis and a PD-L1 negative brain metastasis.

The median number of previous treatment lines was 1 (IQR 0–3). The median duration on previous treatment lines – if applicable – was 4.8 months (IQR 1.8–9.7).

**Fig. 1:**
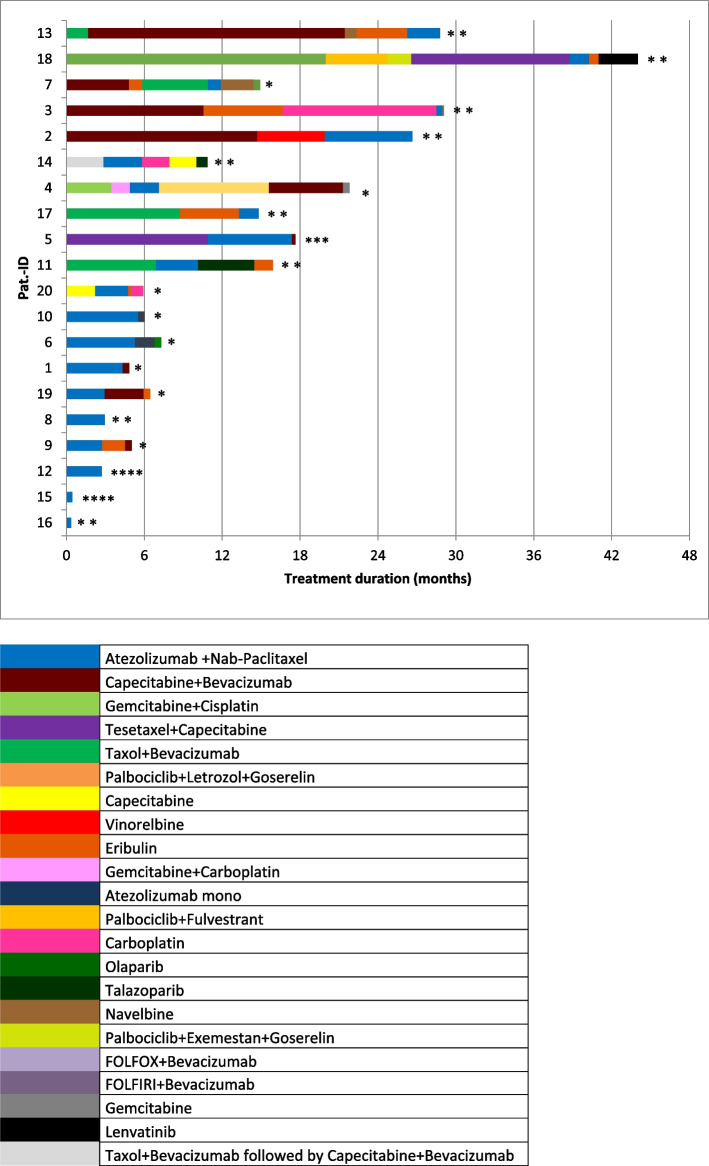
Treatment duration of atezolizumab and nab-paclitaxel, previous and subsequent treatment lines. * ongoing treatment, ** death, *** best supportive care, **** loss to follow-up

Treatment lines prior and subsequent to atezolizumab (and nab-paclitaxel) administration are displayed in Fig. 1.

The median number of received cycles of atezolizumab and nab-paclitaxel was 3.5 (IQR 2.0–5.0). Concordantly to IMpassion130, patients continued with atezolizumab monotherapy in case of good treatment response (*n* = 3).

Treatment with atezolizumab was discontinued owing to the following reasons: progressive disease (11 patients), adverse events (1 patient with thrombocytopenia, 1 patient with hypophysitis, 1 patient with diarrhea), death (in total 3 patients, 1 patient with death owing to adverse event (pneumonitis)) and unknown reason (2 patients).

The median TTF was 2.69 months (IQR 1.52; 3.6).

**Fig. 2:**
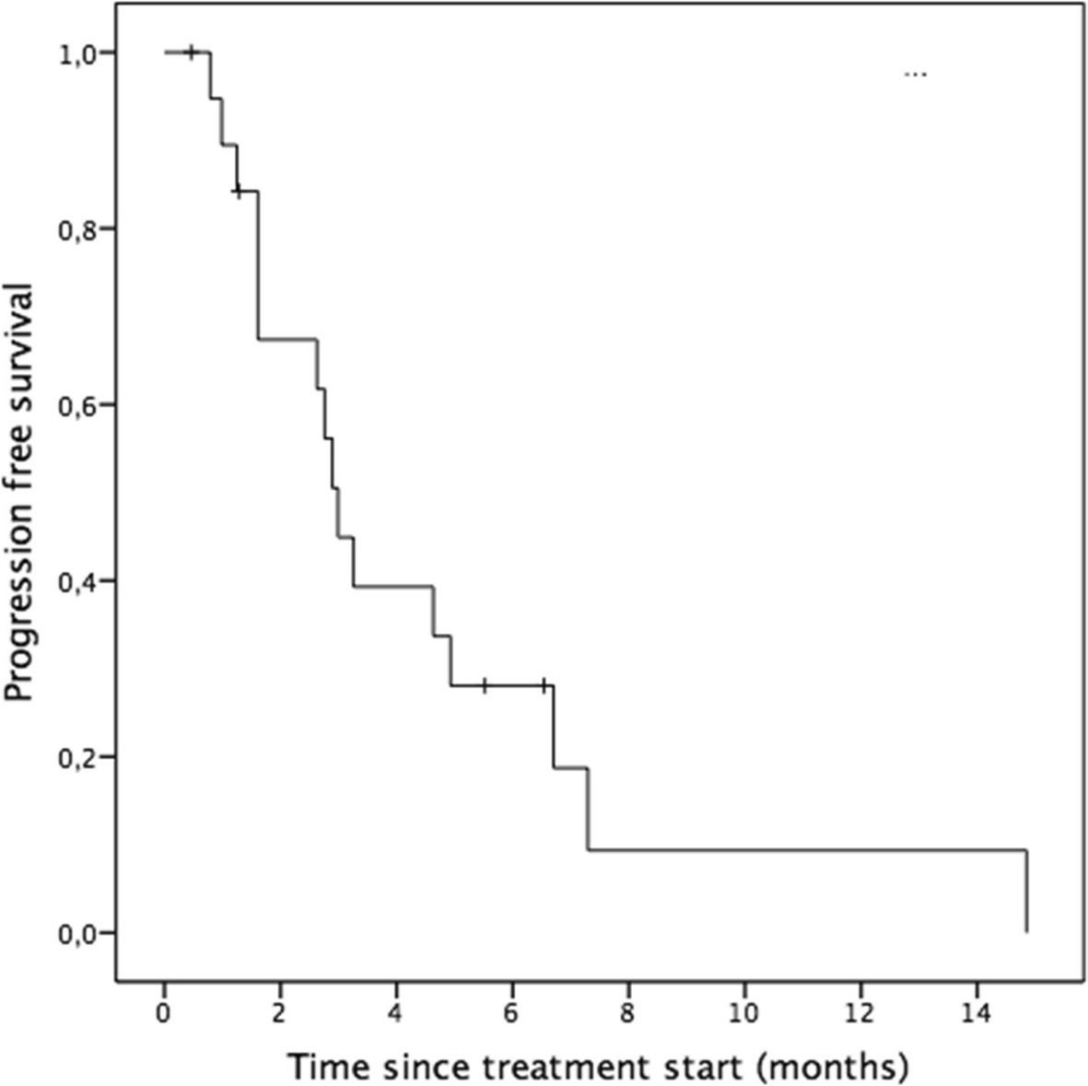
Progression free survival analysis. Number at risk: 3 months: 8 (2), 6 months: 4 (3), 9 months: 1 (4), 12 months: 1 (4)

Median PFS was 3.0 months (SE = .24; 95% CI [2.5;3.5], Kaplan–Meier method) (see Fig. 2). Regarding parameters impacting PFS duration (Mann–Whitney test), the presence of lymph node metastases was significantly associated with a longer PFS compared to no lymph node metastases (*p* = .012). No significant association of PFS was observed for any other tumor location including breast, liver, bone, skin, lung and brain. PFS duration was independent of the number of metastases (r_s_ (*n* = 16) = .01; *p* = .479, Spearman´s rank correlation). PFS showed a marginally significant negative association with the treatment line, with a shorter PFS duration in later treatment lines (r_s_(*n* = 16) = -0.36; *p* = 0.089, Spearman´s rank correlation).

**Fig. 3:**
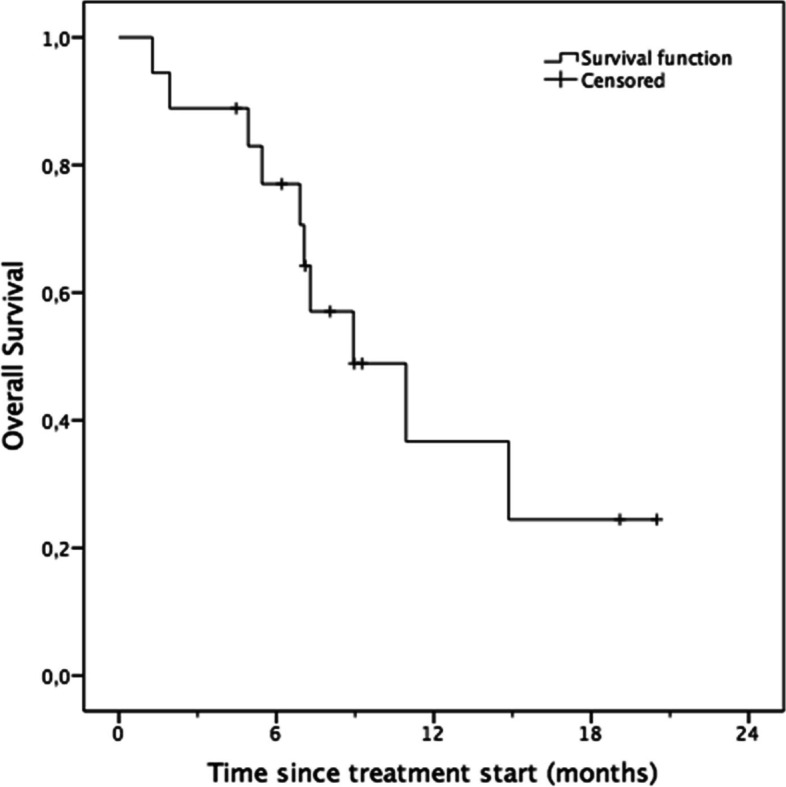
Overall survival analysis. Number at risk: 3 months: 16(0), 6 months: 14 (1), 9 months: 5 (5), 12 months: 3 (6)

Median OS was 8.94 months (SE = 2.34, 95%CI [4.35;13.53], Kaplan–Meier method) (see Fig. 3).

Next, efficacy outcome variables were evaluated separately for patients who received atezolizumab and nab-paclitaxel in the first line treatment setting according to the IMpassion130 inclusion criteria and for patients with atezolizumab and nab-paclitaxel as second line treatment or beyond.

The median time to treatment failure in the first-line treatment cohort was 2.83 months (IQR 2.69; 3.76) compared to 1.61 months (IQR 1.25; 3.25) in the second and beyond treatment line cohort (*p* = .225). When excluding the three patients that also had tissue biopsies with a luminal breast cancer subtype and PD-L1 negative status (in order to assimilate the study population of the present study even closer to the study cohort of the pivotal study IMpassion130) the median time to treatment failure was 2.83 months (IQR 2.69; 3.76) compared to 2.99 months (IQR 1.43; 4.98) in the second and beyond treatment line cohort (*p* = .536).

A negative association between the treatment line and the time to treatment failure was observed, meaning the earlier the patients received atezolizumab and nab-paclitaxel in the course of their palliative treatment the longer the time to treatment failure was (r_s_ = -.48 (*n* = 14), *p* = 0.042). 

Median PFS in the first-line treatment cohort was 4.63 months (SE = 2.28, 95%CI [0.16; 9.10], Kaplan–Meier method) compared to 1.61 months (SE = .72; 95%CI [.20; 3.02], Kaplan–Meier method) in the second and beyond treatment line cohort (*p* = .090). When excluding the three patients that also had tissue biopsies with a luminal breast cancer subtype and PD-L1 negative status median PFS in the first line treatment cohort was 4.63 months (SE = 2.28, 95%CI [.16; 9.10], Kaplan–Meier method) compared to 2.99 months (SE = 1.16, 95%CI [.71; 5.27], Kaplan–Meier method) in the second and beyond treatment line (*p* = .233).

Median OS in the first-line treatment cohort was 14.85 months (SE = 9.82, 95%CI [≥ .01; 34.10], Kaplan–Meier method) compared to 7.29 months (SE = .28, 95%CI [6.76; 7.83], Kaplan–Meier method in the second and beyond treatment line cohort (*p* = .092). Again, when excluding the three patients that would not have matched the IMpassion130 PD-L1 positive cohort median OS in the first line treatment subgroup was 14.85 months (SE = 9.82, 95%CI [≥ .01; 34.10], Kaplan–Meier method) compared to 7.29 months (SE = .40, 95%CI [6.51; 8.08], Kaplan–Meier method) in the second and beyond treatment line (*p* = .032).

The most common adverse events were leucopenia (7 patients (35%)), fatigue (6 patients (30%)) and abdominal pain (4 patients (20%)). Other adverse events included (in descending frequency) hyper- and hypothyreoidism (3 patients (15%)), anaemia (2 patients (10%)), arthralgia (2 patients (10%)), constipation (2 patients (10%)), dyspnoe (2 patients (10%)), headache (2 patients (10%)), neutropenia (2 patients (10%)), peripheral neuropathy (2 patients (10%)), pyrexia (2 patients (10%)), back pain (1 patient (5%)), diarrhea (1 patient (5%)), dizziness (1 patient (5%)), hypophysitis (1 patient (5%)), nausea (1 patient (5%)), pain in extremity (1 patient (5%)), peripheral oedema (1 patient (5%)), pneumonitis (1 patient (5%)), rash (1 patient (5%)), thrombocytopenia (1 patient (5%)) and thrombocytosis (1 patient (5%)).

The safety summary is outlined in Table 2.

**Table 2:** Safety summary

	**IMpassion130**, ITT population, atezolizumab + nab-paclitaxel (*N* = 453)	**Deutschmann C**, et al. (*N* = 20)
Total number of reported adverse events of any cause	7741	50
All-cause adverse events (any grade)	450 (99%)	18 (90%)
Any-grade adverse events of special interest	262 (58%)	8 (40%)
Adverse events leading to discontinuation of any study drug	74 (16%)	6 (30%)
Adverse events leading to discontinuation of atezolizumab (or placebo) only	30 (7%)	0
Adverse events leading to discontinuation of nab-paclitaxel only	74 (16%)	0
Adverse events leading to dose reduction or interruption of nab-paclitaxel	196 (43%)	7 (35%)
Adverse events of special interest leading to atezolizumab (or placebo) discontinuation	9 (2%)	2 (10%)
Treated with systemic corticosteroids within 30 days of onset of the adverse event of special interest	62 (14%)	1 (5%)
Treatment-related deaths	2 (< 1%)	1 (5%)

## Discussion

The present study evaluated the efficacy and safety of atezolizumab and nab-paclitaxel in a real world-setting in 20 metastatic breast cancer patients treated at an academic center in Austria in the initial phase after treatment approval.

At a median follow-up of 7.1 months (IQR 5.2–9.1) median PFS was 3.0 months (SE = .24; 95% CI [2.5; 3.5]) and median OS was 8.94 months (SE = 2.34, 95%CI [4.35; 13.53]).

In comparison, the pivotal study IMpassion130 reported a final median progression-free survival of 7.5 months (95% CI 6.7–9.2) with atezolizumab plus nab-paclitaxel in the ITT PD-L1-positive population at a median follow-up of 12.9 months and an updated median progression-free survival of 7.5 months (95% CI 6.7–9.2) at a median follow-up of 18.5 months (IQR 9.6–22.8), respectively [1, 2]. The median exploratory overall survival at the second interim analysis was 25.0 months (95% CI 19.6–30.7) with atezolizumab plus nab-paclitaxel in patients with PD-L1 positive tumors.

The considerable deviations in efficacy outcome parameters reported in IMpassion130 compared to the present study may be explained by the usage of atezolizumab in later treatment lines, a study population with higher morbidity at baseline and inclusion of tumors with differing biologic characteristics compared to IMpassion130.

While IMpassion130 solely included previously untreated unresectable, locally advanced or metastatic triple-negative breast cancer patients, the majority of patients in the present study had received chemotherapy for metastatic disease prior to atezolizumab and only 45% of patients received atezolizumab and nab-paclitaxel as first line treatment in the metastatic setting. Concordantly to our findings, previous data on atezolizumab monotherapy [3] and pembrolizumab [4] in triple negative metastatic breast cancer patients showed reduced clinical benefit from the anti-PD-L1/PD-1 agents in later lines of therapy. Therefore, immunotherapy should be introduced in the first treatment line for advanced breast cancer as stated in the EMA regulatory approvals [5].

IMpassion130 excluded patients with rapid disease progression referring to patients who had received previous radiotherapy or chemotherapy in the curative setting less than 12 months before randomisation [1]. Contrary, the present study included patients with more aggressive tumors indicated by the considerably shorter mean time from last-surgery until diagnosis with unresectable locally advanced or metastatic disease (9.0 months (6.2–22.5) vs. 24.5 months (15.9–38.9)). Furthermore, the present study population contained a higher proportion of patients with liver (40% vs. 28%) and brain (20% vs. 7%) metastases and – contrary to IMpassion130 – also included 3 patients with treated, yet symptomatic brain metastases. Previous studies on atezolizumab monotherapy for triple-negative metastatic breast cancer patients have demonstrated worse clinical outcomes in the presence of liver metastases regardless of treatment line [3]. Similar results were shown for pembrolizumab in patients with melanoma and non-small cell lung cancer [6]. These findings might be explained by the low prevalence of PD-L1 IC owing to the intrinsic immunosuppressive microenvironment of liver metastases [7, 8]. In addition, a significantly lower number of TILs was observed in breast cancer brain metastases compared to primary breast cancers, also suggesting an immunosuppressive microenvironment [9].

Notably, the IMpassion130 study did not show worse clinical outcomes in exploratory analyses of the subgroup of patients with liver metastases [10].

IMpassion130 demonstrated particular efficacy of atezolizumab and nab-paclitaxel in patients with triple-negative PD-L1 positive tumors exclusively leading to drug approval in this specific patient population.

Contrary, the present study contained three patients with tissue samples with variable immunohistochemistry profiles – with one of them also only showing a positive PD-L1 expression of tumor not immune cells. Additionally, one patient had a mixed PD-L1 expression in different tissue samples.

The predictive value of PD-L1 for PD-L1-targeted therapy in the early and metastatic breast cancer setting has been demonstrated in various studies [11–14]. Moreover, the clinical activity of atezolizumab has been shown to increase with rising IC and TC expression level of PD-L1 in various cancer types [3, 15, 16]. Contrary, in the exploratory biomarker evaluation of the IMpassion130 study the PD-L1 IC + expression level (≥ 1%) did not appear to affect atezolizumab and nab-paclitaxel efficacy as improved PFS and OS hazard ratios were comparable in patients with PD-L1 IC + low (≥ 1% and < 5%) or PD-L1 IC + high (≥ 5%) status [17]. Similarly, Hoda et al. [18] reported of a treatment response to atezolizumab and nab-paclitaxel of two patients with PD-L1 positive primary breast tumors but discordant PD-L1 negative metastatic lesions. Treatment responses of PD-L1 negative various cancer types with PD-L1 targeted therapy have been reported likewise, though response rates in PD-L1 positive tumors were higher [19]. Notably, in a biomarker analysis of atezolizumab monotherapy patients with tumors with a positive (at least 1%) baseline PD-L1 expression on tumor cells but < 1% PD-L1 expression on immune cells were nonresponders.

Eventually, the PD-L1 IC negative and solely PD-L1 TC positive metastatic lesions in the present study, respectively, might have contributed to the worse efficacy outcome compared to IMpassion130. Yet, due to the retrospective nature of the present study and the small sample size an organ-site and PD-L1-expression dependent treatment response evaluation was not feasible.

The median time between the date of tissue sampling that was (later) used for PD-L1 assessment and the start date of atezolizumab and nab-paclitaxel treatment was 6.6 months (1.1–12.4). Owing to the long gap between testing for PD-L1 and treatment initiation it is possible that some tumors lost PD-L1 positivity meanwhile. Indeed, in one out of four patients with more than one available tissue sample, discordant PD-L1 results were obtained. Temporal as well as spatial heterogeneity of PD-L1 positivity in breast cancer has been described in various studies with inconsistent results and needs further evaluation [7, 20–27].

In IMpassion130 PD-L1 expression was tested by immunohistochemistry using the SP142 PD-L1 immunohistochemical assay (Ventana Medical Systems, Oro Valley, AZ, USA) [1]. In the present study the majority of samples were analyzed using the same analytical method, yet in 2 samples the Clone BSR90 (Nordic Biosite) was utilized to assess PD-L1 status. It has to be noted, that published data indicates that different PD-L1 tests are not analytically equivalent and that patient outcomes have the potential to vary depending upon the assay used to identify patients for treatment [28]. SP142 should preferably be used to identify patients eligible for atezolizumab and nab-paclitaxel treatment.

Safety results in the present study were similar to IMpassion130. At the second interim analysis of IMpassion130 the most common grade 3–4 adverse events were neutropenia (38 [8%] of 453 patients in the atezolizumab group), peripheral neuropathy (25 patients [6%]), decreased neutrophil count (22 patients [5%]) and fatigue (17 patients [4%]) [2]. In comparison, the most common adverse events in the present study were leucopenia (7 patients (35%)), fatigue (6 patients (30%)) and abdominal pain (4 patients (20%)).

At the second interim analysis of IMpassion130 adverse events of special interest had occurred in 262 of 453 patients (58%). Similarly, in the present study any-grade adverse events of special interest occurred in 8 of 20 patients (40%).

Treatment-related deaths were reported in two (< 1%) patients in the atezolizumab group (autoimmune hepatitis related to atezolizumab [*n* = 1] and septic shock related to nab-paclitaxel [*n* = 1] at the second interim analysis of IMpassion130. In the present study treatment-related deaths were reported in 1 of 20 patients (5%) (pneumonitis related to atezolizumab).

Furthermore, similar results regarding treatment adherence were observed in the present study compared to IMpassion130.

In IMpassion130 406 of 453 patients discontinued atezolizumab owing to the following reasons: disease progression (*n* = 330), adverse event (*n* = 30), withdrawal by patient (*n* = 17), symptomatic deterioration (*n* = 14), death (*n* = 6), physician decision (*n* = 5), other reasons (*n* = 3) and non-compliance (*n* = 1). Leading cause for treatment discontinuation of atezolizumab and nab-paclitaxel due to toxicity was peripheral neuropathy (20 patients [4%]).

In comparison, in the present study atezolizumab was discontinued owing to the following reasons: progressive disease (11 patients), adverse events (1 patient with thrombocytopenia, 1 patient with hypophysitis, 1 patient with diarrhea), death (in total 3 patients, 1 patient with death owing to adverse event (pneumonitis)) and unknown reason (2 patients).

The following limitations of the present study have to be reported.

The study contained only a small sample size. To support the understanding of the impact of this combination outside of the clinical trial setting the presented findings should be reviewed in consideration of other real-world datasets. Radiologic response evaluation was performed less standardized and frequently compared to IMpassion130 potentially delaying the diagnosis of disease progression and prolonging continuation of treatment. Due to the retrospective nature of the present study and consequentially the inpart lack of clinical data some safety data and secondary efficacy endpoints such as the proportion of patients achieving an objective response, the duration of response and the time to deterioration in global health status evaluated in IMpassion130 could not be assessed in the present study.

In conclusion, the present study—evaluating treatment with atezolizumab and nab-paclitaxel in unresectable locally advanced or metastatic breast cancer patients in a real-world setting—showed a considerably shorter PFS (3.0 vs. 7.5 months) and OS (8.94 vs. 25.0 months) compared to the pivotal study IMpassion130. This might be explained by the inclusion of tumors with differing biologic characteristics compared to IMpassion130, a study population with higher morbidity at baseline and the usage of atezolizumab in later treatment lines. Yet, safety and treatment adherence results were similar between both studies.

## Data Availability

The datasets analysed during the current study are available from the corresponding author on reasonable request.
